# What Human Planning Can Tell Us About Animal Planning: An Empirical Case

**DOI:** 10.3389/fpsyg.2020.00635

**Published:** 2020-04-03

**Authors:** Gema Martin-Ordas

**Affiliations:** Department of Psychology, University of Stirling, Stirling, United Kingdom

**Keywords:** planning, tool use, sequence, preschoolers, adults

## Abstract

The ability to think about and plan for the future is a critical cognitive skill for our daily life. There is ongoing debate about whether other animals possess future thinking. Part of the difficulty in resolving this debate is that there is not a definite methodology that allow us to conclude that animals (and human children) are truly thinking about a future event. Research with humans—both children and adults- will benefit the field of comparative psychology by providing information about the range of humans’ responses when they are faced with problems similar to those presented to other animals. Inspired by a problem that chimpanzees experienced in the wild, children of 4 and 5 years of age and young adults were presented with a situation in which they were expected to select two tools in order to obtain a reward. More older children than 4 years old successfully obtained the reward. Adults also succeeded at solving the problem. However, both children and adults struggled to select the two correct tools *before* any tool-use action was executed. While children’s performance is discussed in the context of temporal components required to envisage future events, adults’ performance is interpreted in the context of cognitive effort. These findings link developmental and adult cognition with comparative psychology.

## Introduction

There is no doubt that humans can think and plan for the future (e.g., Suddendorf and Corballis, 2007). In fact, we spend an important part of our time mind-wandering about the future (e.g., Smallwood et al., 2009; Baird et al., 2011). Developmental research has shown that the ability to think about the future develops between ages 4 and 5 (e.g., Russell et al., 2010; Suddendorf et al., 2011; Atance and Sommerville, 2014). The experimental approach to study future thinking in children has mainly relied on the use of the Spoon test (Tulving, 2005). This test is based on the following scenario: A young girl dreams that she is at a party where all the guests are being served chocolate pudding. To eat the pudding, the young girl needs a spoon, but she does not have one. That night, she falls asleep while holding a spoon. Bringing the spoon represents an instance of future thinking because it implies *envisioning* a need that will occur in the future.

Suddendorf et al. (2011) adapted Tulving’s idea by presenting children with a problem (e.g., locked box with no key) in room A and a set of items (including a key) in room B. Their study showed that 4 but not 3 year olds choose the correct item to take back to room A (for similar results see: Atance and Meltzoff, 2005; Russell et al., 2010; Redshaw and Suddendorf, 2013; Scarf et al., 2013; Atance and Sommerville, 2014; Atance et al., 2015; Cuevas et al., 2015; Dickerson et al., 2018). Overall, these studies typically show an age-related improvement in future thinking between ages 3 to 5. The Spoon test has also been successfully implemented in studies with great apes. For example, Mulcahy and Call (2006) presented orangutans and bonobos with an out-of-reach reward and with a set of useful and useless tools, which they could take into a waiting room. To obtain the reward, subjects had to return to the room where the out-of-reach reward was placed, carrying the useful tool, either 1 or 24 h after having seen the reward. Mulcahy and Call showed that great apes did select and save the correct tool for a future use (see Osvath and Osvath, 2008 for similar results).

However, an important concern with the just described Spoon tests is that it is unclear whether thinking about a *future event* is needed when making successful choices. In these tasks, selecting the correct item may only indicate that subjects know that, for example, the key is useful for unlocking the box *now* without having to represent its use in a future event (e.g., Martin-Ordas, 2018; Hoerl and McCormack, 2019). To address this issue, it is required to demonstrate that individuals have some understanding of what the future might entail. Including a temporal component [i.e., before-and-after relationships; henceforth “temporal reasoning” (McCormack and Hoerl, 2011; Hoerl and McCormack, 2019)] will help assessing when in development the ability to envision the future emerges. Recently, Martin-Ordas (2018) addressed this issue by presenting 3-, 4- and 5-year-olds with a task in which, to secure a future need (e.g., play with a marble run game), children *first* had to obtain a key that allowed them *next* to access the marbles. By the age of four children selected the key; however, it is only by the age of 5 that children reasoned about the temporal sequence of future events *and* selected the key. Thus, this study highlighted the importance of assessing the temporal component of future thinking.

Interestingly, chimpanzees at the Goualougo Triangle (Republic of Congo) have been described to use two tools in sequence—a puncturing stick first and fishing probes next-when trying to access the termites from subterranean nests. Chimpanzees usually arrive at the nests with the two tools and, crucially, they have never been observed to only transport the puncturing stick—alone it would not be effective (Sanz et al., 2004). This study nicely illustrates how planning (e.g., Hayes-Roth and Hayes-Roth, 1979; Miller et al., 1960) might entail a temporal component since transporting *both tools*—as opposed to only bringing one tool regardless of its function—indicates envisioning the *two steps* of the termite-extracting problem (Byrne et al., 2013).

Inspired by Sanz et al.’s (2004) study, a termite-extracting problem was adapted to determine whether children 4 and 5 years of age and adults can plan for a future event that involves selecting two tools. In the current studies, participants were presented with a task that needed a “puncturing” tool to first make a hole on the top a cylinder and a “hook” tool to subsequently pull a reward through the hole (see Weir et al., 2002; Beck et al., 2011; for a similar task). In order to succeed, participants had to envision the two steps of the problem and select the correct two tools. There was a “Spatial-displacement group” (i.e., the task and four items—the two necessary tools and two other functionless items- were placed in two different rooms) and a “No-spatial-displacement group” (i.e., items and task were placed in the same room). For the Spatial-displacement group, successful performance required selecting the tools while holding a memory of the task and envisioning the correct sequence of tool-actions. By comparison for the No-spatial-displacement group, succeeding entailed selecting the tools while only envisioning the correct sequence of actions as the task was in plain view. The human ability to think about the future is unquestioned, thus older children and adult humans should be able to envision the steps of the task and plan accordingly. Note that participants were not trained in the task nor they were given demonstrations on how to solve a functionally equivalent task. In addition, single-trial methods were used in the present studies (e.g., Suddendorf and Corballis, 2007; Suddendorf et al., 2011). As a result, these experiments serve as a potentially interesting test of human planning under the criteria previously defined for animals. In this regard, these findings will contribute to the comparative research not only by offering insights on the range of responses that can be performed in planning tasks but also by identifying under which conditions humans produce those responses.

## Experiment 1: Children (I)

### Materials and Methods

#### Participants

A total of 60 children were recruited, with 1 participant being excluded due to experimental error, resulting in a final sample of 59 participants (28 females; 31 males) aged 4 (*M* = 53.13 months, SD = 2.97, *n* = 30) and 5 (*M* = 65.38, *SD* = 3.44, *n* = 29). All participants were predominantly White, middle class, and fluent in English. Children were tested individually at the Center for Life in Newcastle (United Kingdom). The experiment received ethical approval from the Newcastle University’s Faculty of Medical Sciences Ethics Committee (Project name: Future thinking in children and adults). Parents provided written informed consent for their children’s participation, and children also provided their verbal assent.

#### Procedure

**FIGURE 1:**
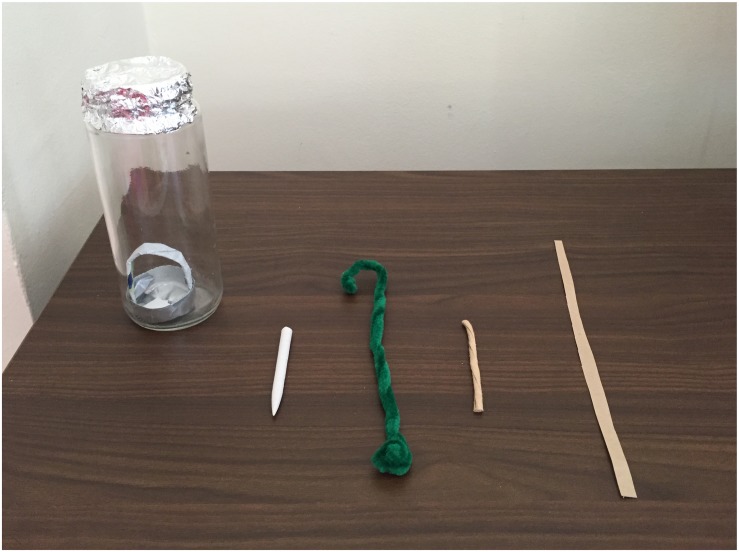
Task and tools used in the present experiment. From left to right: Puncturing tool, hook tool, short tool, and long tool.

The experiment took place in two different areas: Room 1 and Room 2. First, participants were presented in Room 1 with a long narrow transparent container (16 cm length × 4.5 cm diameter) so that they could not use their hands to reach its bottom (e.g., 12). A 18 cm pipe cleaner with a hook made at one end (“hook tool”), a 10 cm long × 4 mm width paper blender stump (“puncturing tool”), a 10 cm long × 4 mm width strip of paper (“short tool”) and a 22 cm long × 4 mm width strip of paper (“long tool”) were used as tools. A bucket containing a reward (e.g., 3 stickers) was placed at the bottom of the container. The opening of the container was covered with extra-strong foil paper and children were explained that the foil was glued to the sides of the container (see Figure 1). Note that the puncturing tool could only function as a tool to puncture the foil paper and the hook tool as a tool to lift the bucket (the two ends of the hook were made soft so they could not pierce the foil paper).

The experimenter (E) said “*If you can get the stickers, you get to keep them.*” Each participant was then randomly assigned to one of two following groups:

(1) *Spatial-displacement group:* For this group, the task and the 4 tools were placed in different rooms. E and participant went to Room 2 (i.e., Tool room). From this area participants did not have visual access to Room 1 (i.e., Task room). E presented participants with the tools and said “*You can use some of these things to help you. Can you think what you will need to get the stickers?*” Children were told that they should get the stickers without turning the container. For each tool-choice opportunity, there were no explicit instructions about the number of tools children could choose—they could choose as many tools as they considered necessary. Likewise, E did not inform about the number of opportunities that participants had to choose tools. Once children made their choice, E and child went back to the Task room. Children were allowed to manipulate the tools so they could learn about the properties of the tools before making their choices. The procedure continued as follows depending on children’s choices:
  1.1. Children chose the 4 tools. E allowed them to try to use the tool on the task. If participants tried to use first any of the incorrect tools, E said: “*Oh no, it does not work because we cannot get through the paper.*” Likewise, if children used the correct tool first and tried to use any of the incorrect tools next, E said: “*Oh no, it does not work because we cannot get the bucket.*” Pilot data suggested that children started to get frustrated if they tried to solve the problem more than four times. For that reason, children were given a maximum of 4 tool-use attempts to obtain the reward. At that point, if the child had not chosen and used the puncturing and hook tools in the correct order, E proceeded to get the bucket out of the container using the correct tools and gave the reward to the participant.
  1.2. Children chose one or more of the following tools: hook, long stick or short stick. E allowed them to try the tool(s) on the container. After each tool-attempt, E said: “*Oh no, it does not work because we cannot get through the paper. Let’s go back to the other room and see if there is something else that could help you get the stickers.*” This procedure was repeated a maximum of 4 times. At that point, if the participant had not chosen and used the puncturing and hook tools in the correct order, E proceeded to get the bucket out of the container using the correct tools and gave the reward to the participant.
  1.3. Participants chose only the puncturing tool. As before, participants were allowed to use the tool on the container. Then, E said: “*What do we do next? Can you think what else you need to get the stickers?*” If children did not spontaneously suggest to go back to the Tool room, E said: “*Let’s go back to the other room and see if there is something else that could help you get the stickers.*” If children chose the hook, s/he was allowed to use it to obtain the reward. If participant chose any of the other tools (e.g., short stick, long stick), E followed the procedure described in the previous sections.
  1.4. Participants chose both the puncturing tool and the hook. E allowed them to use both tools on the apparatus to obtain the reward.
(2) *No-spatial-displacement group*: For this group, the container and 4 tools were placed in plain view in the same room. The same procedure as for the Spatial-displacement group was used. Likewise, for participants’ choices E followed the exact same procedure as above, except that she omitted “*Let’s go back to the other room.*” The rationale for having this condition was to assess whether participants could solve the problem (1) when all the elements of problem were presented in the same room and (2) when the presentation of the task was immediately followed by the presentation of the tools.

#### Data Scoring and Statistical Analyses

Sessions were video-recorded. Participants received a score of 1 if they selected only the two correct tools before using the selected tools for the task (i.e., two-step planning). Any other response (e.g., selecting only 1 tool) received a score of 0. For those participants who only selected 1 tool, which tool was chosen on the first tool-choice opportunity was also scored. Participants were considered to have solved the task (i.e., success = 1) if they obtained the reward by themselves in a maximum of 4 tool use attempts and to fail the task if the E helped them to obtain the reward after 4 attempts (i.e., fail = 0). In addition, the total number of tool-use attempts required to obtain the reward was scored. For example, a child could choose 2 tools (puncturing tool and hook) but scored 3 tool-use attempts (e.g., participant first used hook, then, puncturing tool, then hook). Forty percent of the data was coded by a second rater. Cohen’s *k* for planning and first chosen tool was perfect (*k* = 1.000), and excellent for solving the task (*k* = 0.82).

Pearson chi-square tests were used to analyze the effect of condition and age in planning, task success, and tool chosen first. Kruskal-Wallis tests were used to analyze the effect of age for the total number of tool-use attempts and Mann-Whitney tests were carried out to assess *post hoc* age effects. Cramer’s V, *r*, η^2^, and ϕ were used to report effect sizes for significant effects. Statistical tests were two-tailed, and results were considered significant if *p* < 0.05.

### Results

#### Two-Step Planning

**FIGURE 2:**
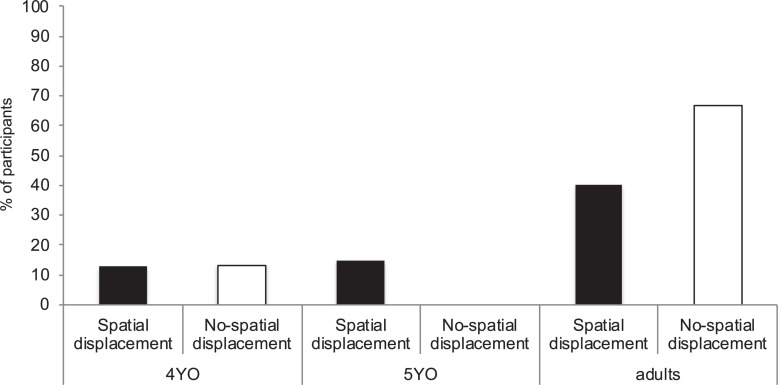
Percentage of 4-year-olds (4YO), 5-year-olds (5YO) (Experiment 1) and adults (Experiment 2) selecting the two correct tools in their first tool-choice opportunity grouped by Spatial-displacement condition and No-spatial-displacement condition.

Overall, age and condition did not have an effect on children’s responses (χ^2^ = 2.66, *df* = 1, *p* = 0.266; see Figure 2). That is, children did not choose the two correct tools in their first tool-choice opportunity either when the tools and task were in the same room (*No-spatial displacement* condition) or when the tools and task were in different rooms (*Spatial displacement* condition). See Table 1 for the percentage of children selecting 1, 2, 3, or 4 tools in both the *No-spatial displacement* condition and the *Spatial Displacement* condition.

**TABLE 1:** Percentage of children and adults selecting 1, 2, 3, or 4 tools in their first tool-choice opportunity in both the *No-spatial displacement* condition and the *Spatial Displacement* condition.

	Experiments 1 and 2							
	Spatial Displacement				No-Spatial Displacement			
	1 tool	2 tools	3 tools	4 tools	1 tool	2 tools	3 tools	4 tools
**4YO**	100%	–	–	–	88%	6%	6%	–
**5YO**	87%	13%	–	–	100%	–	–	–
**Adults**	60%	27%	–	13%	33%	67%	–	–
	**Experiments 3 and 4**							
	**Spatial Displacement**				**No-Spatial Displacement**			
	**1 tool**	**2 tools**	**3 tools**	**4 tools**	**1 tool**	**2 tools**	**3 tools**	**4 tools**
**4YO**	100%	–	–	–	88%	6%	–	–
**5YO**	88%	12%	–	–	100%	–	–	–
**Adults**	20%	47%	20%	13%	20%	74%	–	16%

#### First Tool-Choice

**FIGURE 3:**
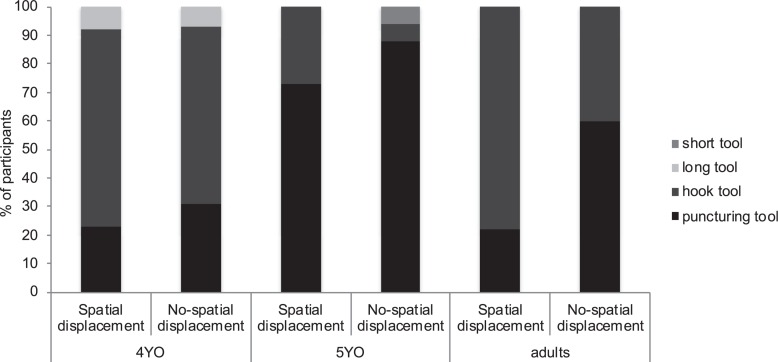
Percentage of 4-year-olds (4YO), 5-year-olds (5YO) (Experiment 1) and adults (Experiment 2) selecting each of the possible tools (i.e., short tool, long tool, hook tool and puncturing tool) in those instances in which they only selected one tool in their first tool-choice opportunity. Data is grouped by Spatial-displacement condition and No-spatial-displacement condition.

Children’s first tool-choice was dependent on age and condition (χ^2^ = 7.30, *df* = 1, *p* = 0.014, *Cramer’s V* = 0.35). Further analyses revealed that for the *Spatial-displacement* condition, selecting the puncturing tool first was not determined by age (χ^2^ = 1.00, *df* = 1, *p* = 0.316). In contrast, age did have an effect for the *No-spatial-displacement* condition (χ^2^ = 7.98, *df* = 1, *p* = 0.008, *Cramer’s V* = 0.52; Figure 3). In this case, more 5 years old selected the puncturing tool first compared to 4 year olds—suggesting that whereas older children might be thinking about the correct sequence in which the problem had to be solved, 4 years old might only be focusing on the last step of the sequence. In fact, from the 5 years old who selected the puncturing tool first, 92% of them selected the hook second.

#### Task Success

Could the above findings be explained by a failure to solve the problem (i.e., obtain the reward)? Age and condition significantly affected participants’ task success (χ^2^ = 17.67, *df* = 1, *p* < 0.001, *Cramer’s V* = 0.55). Fewer 4 years old solved the task compared to 5 years old in both groups (*Spatial-displacement condition*: χ^2^ = 9.94, *df* = 1, *p* = 0.002, ϕ = 0.58; *No-spatial-displacement condition*: χ^2^ = 7.74, *df* = 1, *p* = 0.005, ϕ = 0.51). In fact, whereas 67% of 4 years old in the *Spatial-displacement condition* and 64% in the *No-spatial-displacement condition* obtained the reward, all 5 years old obtained the reward in both the *Spatial-displacement* and *No-spatial-displacement conditions*.

**FIGURE 4:**
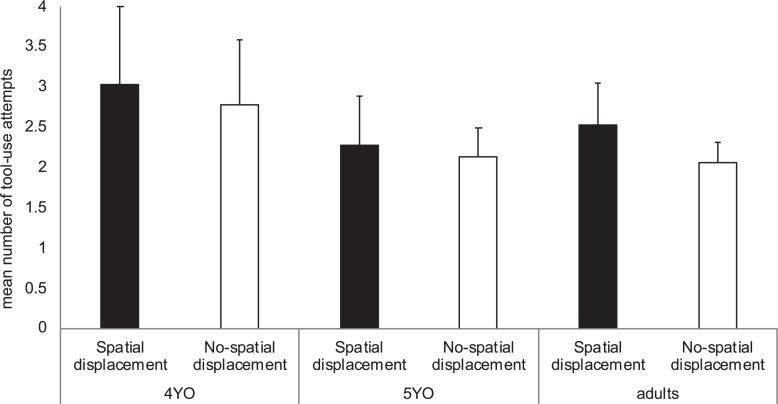
Mean number of tool-use attempts that 4 year-olds (4YO), 5-year-olds (5YO) (Experiment 1) and adults (Experiment 2) needed to obtain the reward grouped by Spatial-displacement condition (black bars) and No-spatial-displacement condition (white bars). Error bars represent the SD.

Age was also found to have a significant effect in the number of tool-use attempts required to obtain the reward. Particularly, 4 years old needed more tool-use attempts to solve the task than 5 years old (*Spatial-displacement condition*: Mann-Whitney: *U* = 63.50, *n* = 29, *p* = 0.032, *r* = 0.54; *No-spatial-displacement condition*: *U* = 64.50, *n* = 27, *p* = 0.044, *r* = 0.57; Figure 4). These results established that poor problem-solving abilities are at play in younger preschoolers’ performance but not in 5 years old.

### Discussion

Pre-schoolers in both *Spatial-displacement* and *No-spatial-displacement* conditions failed to select the two correct tools before performing any tool-use action. More 5-year-old children compared to 4-year-old children solved the task and they did so in fewer tool-attempts than younger children.

Poor problem-solving skills—i.e., failure at sequencing the order in which two tools had to be used- can account for 4-year-old children’s performance. The current findings replicate previous research showing that children’s temporal reasoning abilities are not fully developed before the age of 5 (McColgan and McCormack, 2008; McCormack and Hanley, 2011) and extend them to tool-use tasks involving reasoning about future goals.

There is no question that adults are better at planning than young children. Thus, if the present planning task is still challenging for children because it encompasses envisioning a sequence of actions, then adults would be expected to perform better than children. This possibility was investigated by presenting adults with the same task that children received in Experiment 1.

## Experiment 2: Adults (I)

### Materials and Methods

#### Participants

Thirty-one young adults were recruited and 1 was excluded due to malfunctioning of the apparatus, resulting in a final sample of 30 participants (27 females; 3 males) aged between 18 and 35 years. All participants were predominantly White, middle class, and fluent in English. Participants were tested individually in the lab facilities at the Institute of Neuroscience. The experiment received ethical approval from the Newcastle University’s Faculty of Medical Sciences Ethics Committee (Project name: Future thinking in children and adults). Adult participants provided written informed consent.

#### Procedure

The exact same procedure as in Experiment 1 was followed. Chocolates were used as rewards.

#### Data Scoring and Statistical Analyses

Data coding and statistical analyses were the same was in Experiment 1.

### Results

#### Two-Step Planning

Condition had an effect on participants’ responses (χ^2^ = 4.82, *df* = 1, *p* = 0.028, ϕ = 0.40)—with more participants in the *No-spatial-displacement* condition (67%) choosing the two correct tools before using them than in the *Spatial-displacement* condition (27%) (Figure 2; see also Table 1 for the percentage of participants selecting 1, 2, 3, or 4 tools in both the *No-spatial displacement* condition and the *Spatial Displacement* condition).

#### First Tool-Choice

Participants’ first tool-choice was dependent on condition (χ^2^ = 5.00, *df* = 1, *p* = 0.025, ϕ = 0.40; Figure 3). In this case, more adults selected the puncturing tool first in the *No-spatial-displacement* condition compared to *Spatial-displacement* condition—indicating that having the problem in participants’ view might have facilitated thinking about the sequence in which the problem had to be solved. From those participants who chose the puncturing tool first, 100% chose the hook second in both conditions—indicating that participants might have envisioned the correct sequence of actions.

#### Task Success

Condition did not have a significant effect in the number of tool-use attempts required to obtain the reward (Mann-Whitney: *U* = 112.50, *n* = 30, *p* = 1; Figure 4). Note that all adults succeeded at obtaining the reward in both the Spatial-displacement and No-spatial-displacement groups.

### Discussion

More adults in the *No-spatial-displacement* group compared to the *Spatial-displacement* group successfully chose the correct two tools before performing in the apparatus. All participants in both conditions successfully solved the task.

Certainly, the cognitive mechanisms involved in planning are fully matured in adults. However, adults’ performance in the *Spatial-displacement* group did not select the two tools required to obtain the reward. Motivation or differences in procedure cannot account for these differences because reward and script were the same for both groups. One possibility is that participants in the *No-spatial-displacement* and *Spatial-displacement* groups used different strategies. Decision-making and problem-solving research has shown that adults select among different decision strategies by making a trade-off between the possibility of making correct decisions and the possibility of minimizing effort (Payne, 1976; Johnson and Meyer, 1984; Gigerenzer and Gaissmaier, 2011). For example, when facing a maze problem people usually choose what seems the most direct path to the goal at each step—even though this choice might be incorrect. Similarly, adults in the *Spatial-displacement* group might have traded off accuracy for cognitive effort by selecting the tool that seemingly could have two functions—piercing and extracting^1^. However, in the *No-spatial-displacement* condition such cognitive effort was lessened because task and tools were in plain sight—i.e., not having to recall the task might have facilitated a more effective planning strategy.

In order to investigate this possibility, we presented children (Experiment 3) and adults (Experiment 4) with the same task as before with the difference that now participants were limited to *one* opportunity to choose the tools that they needed to obtain the reward. While it is true that in Experiment 1 children did not show planning behaviors that clearly indicated that they envisioned the two-step sequence, limiting the number of tool-choice opportunities might still prompt them to choose the two correct tools—at least, in older children since they were able to solve the problem. The same could apply to adults. However, if participants are minimizing their cognitive effort, then it would be expected that by limiting to one the opportunity to choose tools, then, at least, adults would select the 4 available tools in the *Spatial-displacement* condition.

## Experiment 3: Children (Ii)

### Materials and Methods

#### Participants

A total of 66 children were recruited, with three participants being excluded due to experimental error, resulting in a final sample of 63 participants (25 females; 38 males) aged 4 (*M* = 55.13 months, *SD* = 3.02, *n* = 32) and 5 (*M* = 64.68, *SD* = 3.03, *n* = 31). All participants were predominantly White, middle class, and fluent in English. Children were tested individually at the Center for Life in Newcastle (United Kingdom). The experiment received ethical approval from the Newcastle University’s Faculty of Medical Sciences Ethics Committee (Project name: Future thinking in children and adults). Parents provided written informed consent for their children’s participation, and children also provided their verbal assent.

#### Procedure

The same materials as in Experiment 1 were used for Experiment 3. The procedure was also the same as in Experiment 1 with exception that when presented with the tools, participants were explicitly told that they could only make a choice: “*Maybe you can use some of these things to help you. But you have to think carefully because you can only choose once, ok? Once you decide what you will need to get the stickers, I will put the things that you did not choose away.*” Once children selected the tool/s, E removed the remaining ones and let the children use the selected ones in the task. For those children who did not choose the correct tools, the E put the non-selected tools back on the table and asked them to obtain the reward by using any of the available tools. As in Experiment 1, children had 4 attempts to obtain the reward. This was done to assess children’s problem-solving abilities. Also as in Experiment 1, 50% of the participants were presented with the task and tools in different rooms (*Spatial-displacement* group) and the other 50% were presented with the task and tools in the same room (*No spatial-displacement* group).

#### Data Scoring and Statistical Analyses

Sessions were video-recorded. Data were coded and analyzed in exact the same way as in Experiment 1. Forty percent of the data was coded by a second rater. Cohen’s k for planning and first chosen tool was perfect (*k* = 1.000), and excellent for solving the task (*k* = 0.90). Cramer’s V, *r*, η^2^, and ϕ were used to report effect sizes for significant effects. Statistical tests were two-tailed, and results were considered significant if *p* < 0.05.

### Results and Discussion

#### Two-Step Planning

Neither age nor condition had an effect on children’s planning behavior (χ^2^ = 0.31, *df* = 1, *p* = 0.573). As in Experiment 1, children did not choose the two correct tools in advance when both the tools and task were in the same room (*No-spatial displacement condition:* 6% of 4YO and none of the 5YO) nor when the tools and task were in different rooms (*Spatial displacement condition:* none of 4YO and 12% of the 5YO; see Table 1). These results suggest that limiting the number of tool-choice opportunities did not improve children’s abilities to select the two tools required to solve the problem.

#### First Chosen Tool

The total number of tools selected by the children in their only choice was not affected by age (Mann-Whitney: *U* = 492.5, *p* = 0.953, *n* = 63) or condition (Mann-Whitney *U* = 470.5, *p* = 0.669, *n* = 63). From all the children who only chose one tool, neither age nor condition were found to affect the selection of the correct first tool (χ^2^ = 2.41, *df* = 1, *p* = 0.121). These results suggest that forcing children to only make one choice did not improve their accuracy at selecting the tools they needed to solve the problem.

#### Task Success

Recall that for all children who did not select the two correct tools in their only tool-choice, E put the remaining objects back on the table. When task success was analyzed, neither age nor condition were found to affect children’s performance (χ^2^ = 0.005, *df* = 1, *p* = 0.941). However, whereas 80% of 5 years old, obtained the reward in both the *Spatial-displacement* and the *No-spatial-displacement* groups, 69% of 4 year-olds did so in both conditions. The number of tool-use attempts was not determined by age (*No-spatial displacement*: Mann-Whitney *U* = 96, *p* = 0.438, *n* = 32; *Spatial displacement*: Mann-Whitney *U* = 103.5, *p* = 0.457, *n* = 31).

Overall, these results replicated the findings from Experiment 1. Younger children’s performance can be explained by their difficulty to solve the problem. In contrast, older children were able to solve the problem but failed to anticipate that they needed two tools to obtain the reward. Next, adults’ performance was examined.

## Experiment 4: Adults (Ii)

### Materials and Methods

#### Participants

Thirty-one young adults were recruited (18 females; 13 males) with one participant being excluded due to experimental error, resulting in a final sample of 30 participants aged between 18 and 35 years. All participants were predominantly White, middle class, and fluent in English. Participants were tested individually in the lab facilities at the Institute of Neuroscience. The experiment received ethical approval from the Newcastle University’s Faculty of Medical Sciences Ethics Committee (Project name: Future thinking in children and adults). Adult participants provided written informed consent.

#### Procedure

The same materials as in previous Experiments were used for Experiment 4. The procedure was also the same as in Experiment 3.

#### Data Scoring and Statistical Analyses

Sessions were video-recorded. Data were coded and analyzed in exact the same way as in Experiment 3.

### Results and Discussion

#### Two-Step Planning

In this case, participants’ planning and ability to envision the two-steps sequence were determined by condition (χ^2^ = 4.88, *df* = 1, *p* = 0.050). In the *Spatial displacement* condition, 46% of the participants selected the two correct tools in their only tool-choice opportunity and 85% did so in the *No-spatial displacement* condition. Thus, compared to Experiment 2, the number of participants selecting the 2 correct tools increased in this Experiment.

#### First Chosen Tool

The total number of tools selected by the participants in their only choice opportunity was not affected by condition (Mann-Whitney *U* = 28.50, *p* = 1, *n* = 30). The idea behind this manipulation was to investigate whether adults were minimizing the cognitive effort by selecting the tool that looked like it could have two functions (e.g., puncturing and lifting). If this were the case, then more participants should have selected the four tools in the *Spatial-displacement* condition compared to the *No-spatial displacement* condition. Note that 20% of the participants chose one tool in both *No-spatial displacement* condition and *Spatial-displacement* condition. In all these cases, participants chose the poking tool first. Moreover, in the *Spatial-displacement* condition 20% of the participants selected three tools and 13% selected the four tools. In the *No-spatial displacement* condition, 16% of the participants selected the four tools and none selected three tools (see Table 1).

Thus, limiting participants to only one tool-choice opportunity increased their tool selectivity although not enough to help them select the two correct tools in the *Spatial-displacement* condition.

#### Task Success

All adults obtained the reward in both the Spatial-displacement and the No-spatial-displacement groups. And the number of tool attempts did not differ between conditions (Mann-Whitney *U* = 97, *p* = 0.508, *n* = 30). As in Experiment 2, these findings demonstrate that performance was not determined by participants’ problem-solving skills.

## General Discussion

The current studies showed that older children and adults were able to *use* two tools in sequence to obtain a reward. Fewer 4-year-old children—compared to older children- did so. Crucially, participants—both adults and children- struggled to anticipate the number of tools required to solve the problem in their first tool-choice opportunity. Although limiting to one the number of tool-choice opportunities improved adults’ performance, children’s responses were not affected by this manipulation. Adults in the *No-spatial-displacement* group successfully selected the two correct tools for the two-step sequence required to obtain the reward, but those in the *Spatial-displacement* group failed to anticipate the two correct tools.

By the age of 2 children have been shown to select an adequate tool based on properties such as length or rigidity (Bates, 1979; Willatts, 1985, 1999; Brown, 1990; Chen and Siegler, 2000; Gredlein and Bjorklund, 2005; see Martin-Ordas et al., 2014 for a study showing that by age 3 children can select a correct tool based on its diameter). In a similar task to the one presented here, results showed that it is only between ages 5 and 8 that children can make a tool suitable to get the bucket out of the tube (Beck et al., 2011; Cutting et al., 2011). Crucially, if children were given a choice between a straight pipe cleaner and a premade hook, by the age of 4 children could select the hook to get the bucket out of the tube (Beck et al., 2011). Thus, children seemed to find difficult the “innovation” aspect of the task (i.e., making the tool), but they understood what properties the tool should have in order for them to obtain the reward (Beck et al., 2011). Importantly, the studies described so far involve using *one* tool to solve a problem. This is in contrast to studies presented here—in which children had to use two tools in a correct sequence of actions to solve the problem. Thus, it is possible that younger children found the current task more difficult than older children did because they lack the ability to sequence the two tool-use actions. This is similar to previous studies showing that it is only by the age of 5 that children can incorporate temporal reasoning to their decision making (e.g., McCormack and Hoerl, 2005; McColgan and McCormack, 2008).

The ability to plan for a future event has been reported to develop between ages 4 and 5 (e.g., Suddendorf et al., 2011; Redshaw and Suddendorf, 2013; Atance and Sommerville, 2014; Atance et al., 2019). In the current experiment, both 4- and 5-year-old children struggled to anticipate the number of tools required to solve the current task. However, their tool selection indicated that children might have been planning for the future event since both age groups tended to select one of the two correct tools (see Figure 3). As mentioned above, lacking the temporal reasoning abilities could account for younger children’s performance to select both tools. However, 5-year-olds did use two tools in sequence to obtain the reward. These findings are in contrast to a previous study showing that by the age of 5 children succeeded in a planning task that required envisioning the order of two future events (Martin-Ordas, 2018). Why did 5-year-old children fail to anticipate the number of tools required to solve the current problem?

There are two crucial differences between Martin-Ordas’ (2018) study and the present ones. First of all, it is possible that whereas in the former the elements of the problem might have been semantically associated (e.g., keys open locks), in the task presented here such semantic association did not exist (e.g., pipe cleaners shaped as hooks might not necessary always be used to lift buckets). Second, whereas in Martin-Ordas (2018) children had to select *one* tool and decide the order in which two future events should happen (e.g., select the key, then visit the marble room to get the marbles and, next, go to the marble room), in the current task children had to envisage the two future actions in order to select the two correct items—with each action being associated to a particular tool. This aspect might have posed more cognitive demands to solve the problem—which, as a consequence, might have increased the difficulty of the task [see Burns and Russell, 2016 for a study showing only children over 5 years of age were able to anticipate a future event when the cognitive demands of the task were high (e.g., spatio-temporal predictions based on someone else’s point of view)].

Adults can plan, envisage the future and think about temporal sequences, so why did they struggle in the current task? The studies presented here indicated that when limiting to one the number of tool-choices opportunities, more participants selected the correct two tools *before* performing any action on the task—although only in the *No-spatial-displacement* condition. These results suggest that adults might be selective planners—that is, even though they can plan, adults might only make use of this ability under particular circumstances. Recent developmental studies have highlighted that performance in the Spoon test is drastically affected when children are asked to *spontaneously* generate the solution to a problem rather than selecting a tool from a number of options (e.g., Moffett et al., 2018; Atance et al., 2019). These studies indicate that it is only by the age of 5 that children start to generate the solutions for a future problem. Along the same lines, the results presented here suggest that limiting participants’ choices to one—and consequently, increasing the costs of making errors- affected their tool selectivity, at least, when the problem was in plain sight. This is similar to what previous tool-use studies with humans (Silva and Silva, 2010, 2012) and great apes (Mulcahy et al., 2005; Martin-Ordas et al., 2012) have already shown.

The constellation of results presented here suggest that, at least, adult humans’ planning responses varied depending on whether the problem was in plain sight compared to when the problem was out of sight. It would be difficult to argue that adults in these experiments did not understand the critical features of the tasks that they had to solve—otherwise the differences in performance between the *Spatial-displacement* and *No-spatial displacement* conditions would not have been found. However, this explanation remains as a possibility for children’s performance. Still, children’s responses in the present tasks do not necessarily indicate an inability to plan. As mentioned earlier, children are not randomly choosing one of the four tools; and their first tool choices seem to indicate that there is a representation of the future event—although, they seem to have difficulties to envision the two steps of the problem.

These limitations should not undermine the value of the present studies. The results presented here still have crucial implications for the field of animal future thinking and planning. In the current studies, participants were presented with an unfamiliar tool-use task. It could have been possible that both children and adults performed better if presented with a more familiar problem (i.e., a task in which a strong semantic association between tools and task existed). However, not all planning situations require dealing with familiar contexts or objects. As such, it is also insightful to study this ability and its flexibility in less accustomed contexts. Additionally, Suddendorf et al. (2011) suggested that tasks aiming to test future thinking should involve using novel problems in order to avoid (associative) learning. This is an important factor to understand future thinking in animals, since, in most cases, subjects are presented with unfamiliar situations that require the use of training and multi-trial methods. In the last 20 years, comparative psychologists have provided empirical evidence that other animals possess some type of future thinking abilities (e.g., Mulcahy and Call, 2006; Osvath and Osvath, 2008; Kabadayi and Osvath, 2017). These findings have been the focus of arduous debates—with some claiming that future thinking abilities in some animals are similar to those in humans (e.g., Martin-Ordas et al., 2014) and others arguing that even the strongest pieces evidence of future thinking in animals can be acknowledged to be no more than (associative) learning achievements (e.g., Suddendorf and Corballis, 2007). Accordingly, if providing subjects with more than one trial (i.e., repeated exposure to the same stimulus-reward relationship) would entail that associative learning—rather than future thinking- could account for their performance, then a first response preferably without training should be considered the standard to show future thinking. Nonetheless, the artificiality of this situation might undermine performance, as one could argue for the present studies—recall that a single-trial method was used to test participants in the present studies. Thus, these studies with children and adults highlight conceptual and methodological issues in the criteria described to asses future thinking (e.g., Anderson, 2001; Silva et al., 2005; for similar arguments on tool-use tasks). The studies also provide the set of responses that humans display under some of the conditions required to test future thinking in animals.

To conclude, more older children and adults compared to younger children succeeded at using two tools in sequence to obtain a reward. Whereas children did not select the 2 tools required to solve the problem in their first tool-choice opportunity, adults were able to do so when the task was in plain view. Human performance in the present tasks highlights important points for comparative research. First, the issue of how to measure future thinking seems to not be completely solved if we are to focus on the novelty of the problems and the lack of training in order to rule out associative learning as the mechanism driving performance in these tasks. Thus, criteria that can equally be applied to humans and animals and that allow us to draw irrefutable comparisons across species are needed. Second, including groups of children and adults in comparative studies will offer reliability to the results and will be informative comparison groups for behavioral tests of these capacities in animals (e.g., Anderson, 2001; Silva et al., 2005; Silva and Silva, 2006). Examining what humans can do will provide us with critical information to be able to identify shortcomings in the study of the comparative research of future thinking and also to provide a context in which to interpret animals’ responses.

## Data Availability Statement

The datasets generated for this study are available on request to the corresponding author.

## Ethics Statement

The studies involving human participants were reviewed and approved by The experiment received ethical approval from the Newcastle University’s Faculty of Medical Sciences Ethics Committee (Project name: Future thinking in children and adults). Written informed consent to participate in this study was provided by the participants’ legal guardian/next of kin.

## Author Contributions

GM-O designed the study, collected the data, and wrote the manuscript.

## Conflict of Interest

The authors declare that the research was conducted in the absence of any commercial or financial relationships that could be construed as a potential conflict of interest.

## Footnotes

1 All adults who chose the hook first attempted to use it to obtain the reward. Additionally, more adults in the *Spatial-displacement* group used the hook first compared to the *No-spatial-displacement* group (χ^2^ = 7.77, *df* = 1, *p* = 0.005).
